# Integrated Approaches for the Delivery of Maternal and Child Health Services with Childhood Immunization Programs in Low- and Middle-Income Countries: Systematic Review Update 2011–2020

**DOI:** 10.3390/vaccines12121313

**Published:** 2024-11-23

**Authors:** Monica P. Shah, Christopher J. Morgan, James G. Beeson, Elizabeth Peach, Jessica Davis, Barbara McPake, Aaron S. Wallace

**Affiliations:** 1 Global Immunization Division, Global Health Center, Centers for Disease Control and Prevention, Atlanta, GA 30333, USA; mshah2@cdc.gov (M.P.S.); ccu7@cdc.gov (A.S.W.); 2 Jhpiego, a Johns Hopkins University Affiliate, Baltimore, MD 21231, USA; 3 Melbourne School of Population and Global Health, University of Melbourne, Melbourne, VIC 3053, Australia; barbara.mcpake@unimelb.edu.au; 4 Burnet Institute, Melbourne, VIC 3004, Australia; james.beeson@burnet.edu.au (J.G.B.); e.peach@unsw.edu.au (E.P.);; 5 Department of Infectious Diseases, University of Melbourne, Melbourne, VIC 3053, Australia; 6 School of Translational Medicine, Monash University, Melbourne, VIC 3800, Australia; 7 Rural Clinical Campuses, University of New South Wales Medicine and Health, Sydney, NSW 2052, Australia

**Keywords:** integration, vaccination, immunization, service delivery, systematic review, maternal and child health, infant, HIV, malaria, family planning

## Abstract

**Background**: The integration of maternal and child health services (MCH) with routine immunization is an important global health strategy, particularly in low- and middle-income countries (LMICs). However, evidence is lacking regarding the best practices for service integration and the effect of integration on immunization and linked health service outcomes. **Methods**: We searched publication databases and gray literature for articles published between 2011 and 2020 that include approaches to integrating MCH services with immunizations during the first two years of life in LMICs. Abstracts and full-text articles were screened for eligibility. For the included articles, data extraction and analysis examined the descriptive characteristics of studies, outcomes, and implementation considerations. **Results**: Among the 16,578 articles screened, 44 met the criteria for inclusion, representing 34 studies, of which 29 were from Africa. The commonly linked MCH services were family planning (24%), human immunodeficiency virus (HIV) diagnosis or care (21%), and malaria prevention or control (21%). Multiple integration strategies were typically used; the co-location of linked services (65%), the provision of extra services by immunization staff (41%), and/or the provision of extra information by immunization staff (41%) were the most common. In general, integration improved MCH service outcomes (76%) and was either beneficial (55%) or neutral for immunization (35%), with some examples in family planning, malaria, and HIV where integrated services were not beneficial. Important implementation considerations included the careful matching of target populations in service re-design, ensuring support from policy, logistics, and information systems, the provision of adequate training and support of staff to avoid overload, clear client communication regarding service integration, and the need to address community concerns. **Conclusions**: Integrating MCH services with routine immunization can expand linked services and improve immunization coverage. This study has identified key implementation considerations relevant to both childhood and adult vaccination programs. More research is needed regarding costs and client preferences.

## 1. Introduction

The integration of other health and development interventions with immunization services has long been viewed as a cornerstone of global immunization strategies. The latest global framework, known as Immunization Agenda 2030 (IA2030), states the strategic priority of ensuring that all people benefit from recommended immunizations, integrated with essential health services, across the life course [1]. Integrating other health interventions with immunization services can contribute to the goal of universal health coverage, where all people have access to the full range of quality health services they need, when and where they need them [2].

Several rationales exist for integrating other health interventions with immunization services. Providing a package of care in a single visit to eligible individuals and accompanying family members can address individual health needs more comprehensively than vertical or single-disease-focused practices, while also reducing the individual’s time and financial costs of seeking care. Childhood immunization services have among the highest and most equitable coverage of any childhood health service [3]. Therefore, immunization visits may provide an important platform for increasing the coverage of other health interventions, particularly in low- and middle-income countries (LMICs) where the coverage of other health interventions may be substantially lower. Integration has the potential to also increase health system efficiency, which can facilitate expanded program reach. Increased efficiency and reach can reduce mortality and morbidity from both vaccine-preventable and non-vaccine-preventable diseases.

However, integrating services is not without concern, as a lack of sufficient investment could lead to overburdened health workers and a decreased quality of service, with potential impacts on service coverage and acceptability [4]. Additional concerns exist regarding the feasibility of service integration due to differing service delivery requirements for considerations such as privacy, delivery time, and the target age range [5,6]. 

To inform global recommendations, 2 previous systematic reviews, published in 2009 and 2012, comprehensively examined 59 studies integrating maternal and child health (MCH) interventions with immunization services to synthesize the lessons learned and best practices [4,6]. The reported outcomes in the studies reviewed indicated the need for increased coverage for the linked services, although not necessarily to the same levels as seen for immunization services. However, most studies did not report outcomes for immunization services. The reviews noted continuing evidence gaps regarding the cost efficiency of integrating services and the impact of integration on immunization outcomes. Despite these evidence gaps, these reviews provided sufficient evidence to support the development of the 2018 World Health Organization (WHO) technical guidance on the integration of immunization services, entitled “Working Together: an integration resource guide for immunization services throughout the life course” [7]. This WHO guidance, aimed at national immunization programs within Ministries of Health, provides an overview of integration approaches and opportunities, as well as considerations of how to best integrate other health services with immunizations. 

Since 2012, when the last systematic review on this topic was published [6], the interest in integrating services has continued to increase, due in large part to a focus on achieving universal healthcare. The 2023 Lusaka Agenda outlines a framework for how global health initiatives (GHIs), which generally focus on supporting a single health program such as immunization, can support a more coordinated approach to strengthening primary healthcare in the integrated delivery of services at a high and equitable level of coverage [8]. Additionally, since 2012, new vaccines have been introduced, and many LMICs have additional doses in the second year of life, providing new opportunities to examine how to integrate health services for these age groups [9]. Meanwhile, efforts to ‘catch children up’ on earlier missed vaccines—such as the Big Catch-Up during the COVID-19 pandemic—have focused on how to opportunistically integrate immunization into other health services [10]. This new context places a greater emphasis on understanding how best to integrate immunization services, tracking the latest evidence that has emerged across studies. 

To inform global, regional, and country-level strategies with the new evidence on the integrated delivery of MCH interventions with immunization services, we undertook an update from the last systematic review on this topic, which reflected studied published up through 2010 [6]. In this review update, we synthesized published and unpublished literature from 2011 to 2020 on integrated approaches for the delivery of MCH services along with childhood immunization programs in LMICs. The objectives of this review were to describe the types of intervention, immunization service delivery platform (fixed, outreach, campaign, mixed), and service change (such as the co-location of services, the provision of extra information or counseling provided, the provision of extra services) used to integrate other services with immunization services provided in the first two years of life. We qualitatively summarized the effect of integration on linked MCH service and immunization outcomes (if measured and reported) and described implementation considerations for integrated service delivery changes. 

## 2. Materials and Methods

The methodology used to conduct this systematic review update draws from the standardized guidelines developed by the Cochrane Collaboration’s Effective Practice and Organization of Care group [11]. Detailed information about the methods is described in a protocol registered with the International Prospective Registry of Systematic Reviews [12]; a brief summary is provided here. 

### 2.1. Search Methods

**Electronic searches:** The following databases were searched on 13 May 2016; 15 May 2020; and 10 March 2023 for articles published between January 2011 and June 2020 (covering the next decade since the previous review [6]) in the English language using a search strategy combining terms for service types (e.g., antenatal, malaria, vitamin A, etc.), similar to a previous review on this topic [6], with additional search terms using integration terminology (full list of terms provided in PROSPERO protocol CRD42016045876 [12]: MEDLINE (via Ovid), EMBASE (via Ovid), CABI Global Health (via EBSCOhost), CINAHL (via EBSCOhost), Cochrane Central Register of Controlled Trials, and the Cochrane Database of Systematic Reviews. **Searching other resources:** Additional publications were identified from the gray literature search of documents from the following agencies: World Health Organization (WHO), UNICEF, BASICS, Family planning—EPI integration working group, Knowledge4Health, POPLINE, John Snow Incorporated, Family Health International, USAID-supported programs, DfID, and the Nigerian Urban Reproductive Health Initiative. 

### 2.2. Criteria for Inclusion and Exclusion

All study designs reporting empirical data were included, while articles from mathematical modeling studies were excluded. Additionally, expert opinions (e.g., perspective articles) and studies that did not focus on the operational impact of integrated service delivery were excluded. Other systematic reviews were excluded, although their reference lists were reviewed to identify any additional eligible studies. We included studies that integrated health services with childhood immunization programs for children aged two years or younger. The review was limited to data from LMICs, as defined by the World Bank for 2016 [13]. Studies were eligible for inclusion if they included one or more linked service and one or more immunization service that had been integrated during pregnancy, postnatally, or in childhood (including the first two years of life) as a function of health service innovation. Studies reporting on missed opportunities for vaccination, where the contact point for integration was health services other than immunization delivery, were not included in this review.

We included studies that involved comparisons with a non-integration population group or pre–post integration comparisons. We excluded studies that had no comparator population group or timeframe. For inclusion in outcome synthesis, studies had to report one or more of the following outcomes for at least one immunization or linked health service: vaccination coverage, vaccination drop-out rates (e.g., first dose of diphtheria-tetanus-pertussis-containing vaccine [DTP1] to DTP3, first dose of pentavalent vaccine [DTP + hepatitis B + Haemophilus influenzae type B; penta1] to penta3), the coverage of a linked MCH service, the number or uptake of services provided (e.g., doses administered, number of tests administered, etc.), the quality of care, the equity of access or coverage, or costs.

### 2.3. Selection of Studies

Two review authors screened all titles and abstracts for inclusion and assessed full-text articles for eligibility. For studies included at the full-text stage, two review authors extracted data from each article in a pre-tested standardized excel template. Any discrepancies were resolved through discussion or by a third reviewer. Covidence systematic review software (Veritas Health Innovation; Melbourne, Australia) was used to manage the independent assessment of articles across authors at the title/abstract and full-text stages. 

During title and abstract screening, multiple publications from the same study were included as individual articles. At the full-text stage, multiple articles from the same study were collated as a single study. Additionally, studies that did not meet inclusion criteria at the full-text stage were listed in a table with their reasons for exclusion. The study selection process was summarized using a Preferred Reporting Items of Systematic reviews and Meta-Analyses (PRISMA) diagram [14]. 

### 2.4. Data Extraction

The data extraction template included the following variables: the descriptive characteristics of the study (e.g., geographic location(s), the year of publication, the timing of data collection, etc.); the study design, including comparison groups; the immunization platform selected for integrated service delivery (e.g., either routine or campaign, and further described by fixed, outreach, mobile, or mixed delivery routes); a description of the integration strategy, including the immunization service (e.g., specific vaccines) and additional health service being linked; outcome measures for both the immunization and linked service; and implementation determinants, considering the perspectives of providers of care and recipients of care, health systems, and the social and political context.

The quality of the evidence was assessed using frameworks derived from GRADE assessments [15] for quantitative data and from the Clinical Appraisal Skills Program (CASP) tools [16] for qualitative data. Since meta-analyses were not conducted and the risk of bias was not a consideration affecting inclusion, the results of the risk of bias assessment are not presented here. 

### 2.5. Data Synthesis

Narrative summaries were compiled for key results, following standard practices [17,18], based on the consensus among three authors (C.J.M., M.P.S., A.S.W.). As part of data synthesis, integration strategies were categorized as one or more of the following: a. extra services provided by immunization staff (i.e., testing, treatment, referrals, etc.); b. extra information and/or counseling provided by immunization staff (i.e., one-on-one consultation, providing informational materials, etc.); c. the co-location of services (e.g., same timing or same physical location of immunization delivery and linked health service); d. vaccination provided by non-immunization staff; and e. extra information and/or counseling provided by non-immunization staff (i.e., screening, health education, or immunization promotion, etc.). The results are summarized in a table that is arranged by EPI platform (campaign versus routine), linked health service(s), and study (designated by first author’s last name and year of publication). 

A single qualitative summary was used to summarize the direction of effect of the integration for the primary outcome(s) for immunization and for the linked MCH service separately. The terms used are defined as follows: “not measured”—there was no outcome measured or no non-integration comparison group available; “negative”—there was lower coverage or service provision in the integration compared to the non-integration group; “mixed negative”—some outcomes were lower in the integration compared to the non-integration group, but others remained static; “static”—no meaningful difference in coverage or service provision between integration and non-integration groups; “mixed positive”—some outcomes were higher in the integration compared to the non-integration group, but others remained static; or “positive”—higher coverage or service provision in the integration compared to the non-integration group. The results are summarized in a table that is arranged by EPI platform (campaign versus routine), linked MCH service(s), integration strategy, and study.

A narrative synthesis was undertaken to synthesize study findings related to the implementation considerations that affected service integration. Implementation considerations affecting service integration were collected into pre-defined categories, including policy and governance topics; the design of integrated service delivery; human resources for health; management, logistics, and costs; operational tools, reporting, and recording; and community and client topics [19]. 

Data were managed, summarized, and synthesized in Microsoft Excel. 

## 3. Results

### 3.1. Results of Search

**Figure 1:**
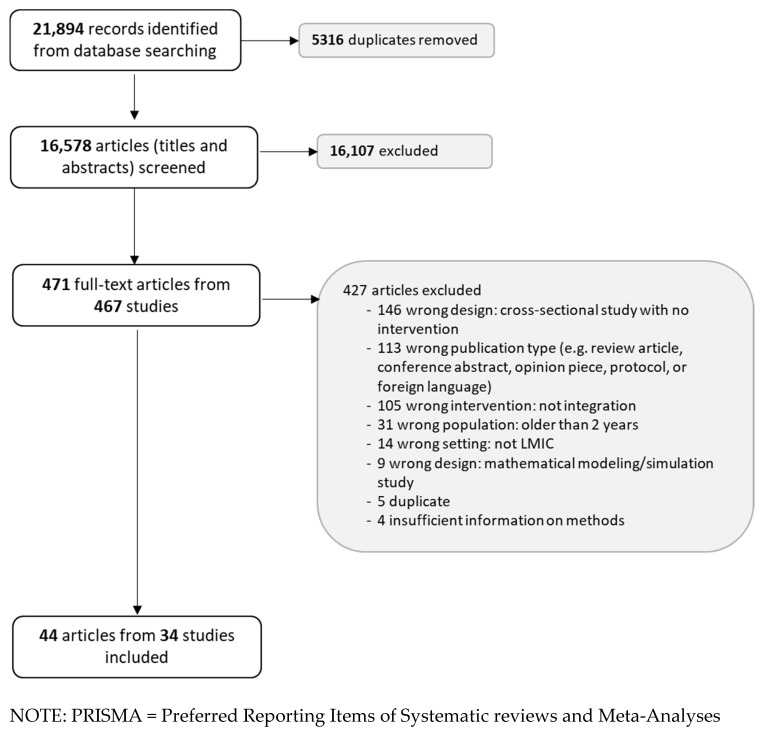
Study flow diagram (PRISMA Diagram), systematic review update 2011–2020.

A total of 21,894 records were identified via database searching and, after removing duplicates, 16,578 titles and abstracts were screened (Figure 1). Among these, 471 full-text articles (from 467 studies) were reviewed and 44 articles (from 34 studies) met the eligibility criteria for inclusion; a full list of full-text articles (*n* = 427) that did not meet eligibility criteria and the reason for exclusion is provided as in the Supplementary Materials as Table S1, List of studies excluded on full-text review and reason for exclusion (*n* = 427). The most common reasons for full-text exclusion were wrong study design (34%), wrong publication type (27%), or articles not reporting on service delivery integration as the intervention (25%). 

At the title and abstract screening stage, reviewers A and B had substantial agreement (Cohen’s kappa: 0.71), while reviewers A and C had fair agreement (Cohen’s kappa: 0.22). At the full-text stage, reviewers A and B and B and C had fair agreement (Cohen’s kappa 0.31 and 0.33, respectively), while reviewers A and C had a level of agreement no better than what would be expected by chance (Cohen’s kappa: 0). 

### 3.2. Description of Included Studies

Of the 34 studies included on integrated service delivery (Table 1, Table 2, Table 3 and Table 4), 29 (85%) were conducted in Africa (Benin, Burkina Faso, Cameroon, Gambia, Ghana, Kenya, Liberia, Malawi, Mali, Madagascar, Nigeria, Rwanda, Sierra Leone, South Africa, South Sudan, Tanzania, and Zambia), 4 (12%) were conducted in Asia (Afghanistan, India, Lao PDR, and Pakistan), and 1 (3%) was conducted in the Americas (Mexico). A majority of included studies reported about the integration of services with routine immunizations (88%) delivered at either health facilities (*n* = 20), outreach sessions (*n* = 3), mobile sites (*n* = 2), or a combination of sites (*n* = 5), while the remaining (*n* = 4, 12%) studies reported about the integration health services within immunization campaigns (Table 1). To summarize further, Table 1, Table 2, Table 3 and Table 4 are grouped by immunization delivery platform and/or linked health service; Table 1 describes studies that were integrated into campaigns and includes 4 studies, performed in Lao PDR, Madagascar, Nigeria, and Pakistan. Table 2 describes studies integrating child health screening or family planning advice and services into routine immunizations and includes 10 studies performed in Benin, Burkina Faso, Ghana, India, Liberia, Malawi, Nigeria, Rwanda, South Africa, and Zambia. Table 3 reports studies integrating HIV and malaria services into routine immunizations and includes 7 HIV studies from Cameroon, Kenya, Malawi, Nigeria, Tanzania, and Zambia, and 7 malaria studies from Gambia, Ghana, Mali, Malawi, Rwanda, and Tanzania. Finally, Table 4 describes the integration of primary healthcare (PHC) services; nutrition, water, sanitation, and hygiene (WASH); or a combination of services into routine immunizations and includes 7 studies conducted in Afghanistan, Kenya, Mexico, Nigeria, Sierra Leone, and South Sudan.

Linked health services included child health, hearing, or vision screening (Table 2); family planning (Table 2); HIV prevention, diagnosis, and treatment (Table 3); malaria prevention, control, and treatment (Table 3); packages of MCH and/or other PHC services, including antenatal care (ANC), prenatal care (PNC), and the integrated management of childhood illnesses (IMCI) (Table 4); nutrition; WASH services (Table 4); or a combination of these (e.g., PHC and nutrition; family planning and nutrition; malaria, PHC, nutrition, and WASH; this is shown in Table 4). Of these, the most commonly reported linked health services were family planning (24%; of which, there were *n* = 7 in Africa and *n* = 1 in India), HIV (21%; of which, there was *n* = 7 in Africa [100%]), and malaria (21%; of which, *n* = 7 in Africa [100%]). Only four studies reported the integration of multiple health services with routine immunization service delivery (Table 4), and this was observed in Bawa, 2019 (malaria, PHC, nutrition, and WASH) [20,21]; Edmond, 2020 (PHC and nutrition) [22]; Habib, 2017 (PHC and nutrition) [23]; and Hodges, 2015 (family planning and nutrition) [24].

The risk of bias was assessed as described in the Materials and Methods Section; most studies were rated as having a high risk of bias due to the implementation science framework of many study designs. As a result, the risk of bias assessment result was not considered as a criterion for inclusion in the review.

**Table 1:** Characteristics of included studies (*n* = 4) on integration into immunization campaigns by linked health service, systematic review update 2011–2020.

Study ^a^	Country (Study Year(s))	Immunization Delivery Platform	Linked Maternal and Child Health Service(s)	Integration Strategy	Description of Integration Strategy	Study Design: Outcome Comparison	Immunization Outcomes Measured (Primary Outcome in Bold)	Linked Health Service Outcomes Measured (Primary Outcome in Bold)
Goodson (2012) and Kulkarni (2010) [25,26]	Madagascar [2007–2008]	Campaign—mixed	Malaria	a. Extra services by immunization staff c. Co-location	Integrated delivery of ITNs, deworming tablets, vit A and measles vaccine to <5-year-olds during a national health campaign.	Post only; two groups [integration = ITNs distributed in campaign package of services; comparison = ITNs were not included]	**Measles vaccination coverage (higher in integration group compared to non-ITN control group, including in poorest households)**	**Proportion of households with at least one ITN (higher in integration compared to non-integration areas)**
Habib (2017) [23]	Pakistan [2013–2014]	Campaign—mixed (fixed and mobile camps)	Nutrition, PHC	b. Extra information and/or counseling by immunization staff c. Co-location	MCH camps alongside polio campaign, offered counseling on hygiene and nutrition, routine immunizations, micronutrient supplements, and general MCH assessments.	Pre/post; three groups: comparison = OPV with routine immunizations; integration 1 = OPV with routine immunizations and other interventions; integration 2 = OPV with routine immunizations, other interventions, and IPV	**-OPV coverage (higher in integration groups versus comparison)** **-Other routine immunization coverage (higher in integration groups versus comparison)**	**Updates of PHC services noted but were not quantifiable due to lack of comparison data.**
Boselli (2011) [27]	Lao PDR [2007]	Campaign—mixed	Nutrition	a. Extra services by immunization staff	Integrated deworming tablets, vit A and polio vaccine to <5-year-olds. Deworming tablets with tetanus toxoid vaccine to women	Only examined cost effectiveness of integrated vit A and deworming tablet delivery compared to non-integrated delivery	**Not measured**	**Not measured**
Birukila (2017) [28]	Nigeria [2013]	Campaign—mixed (fixed and temporary sites)	PHC	c. Co-location	During polio campaigns, health camps were organized for settlements with high vaccine refusal rates in which other health services were offered with OPV.	Historical comparison 2013 with 2014; integration group = settlements with integrated health camps (polio and other primary healthcare services) in 2013.	**Number of children who received OPV (14,000 in 2013 round with integrated health camps and 7000 in 2014 round without integrated health camps)**	**Not measured**

^a^ the last name of the first author and the publication year; studies are ordered by linked maternal and child health service, and then alphabetically by author. Notes: ITN = insecticide-treated bednet; IPV = inactivated polio vaccine; MCH = maternal and child health; OPV = oral polio vaccine; PHC = primary healthcare; vit A = vitamin A supplementation.

**Table 2:** Characteristics of included studies (*n* = 10) on integration of child health or family planning into routine immunizations, systematic review update 2011–2020.

Study ^a^	Country (Study Year(s))	Immunization Delivery Platform	Linked Maternal and Child Health Service(s)	Integration Strategy	Description of Integration Strategy	Study Design: Outcome Comparison	Immunization Outcomes Measured (Primary Outcome in Bold)	Linked Health Service Outcomes Measured (Primary Outcome in Bold)
Integration into routine immunization—child hearing screening								
Friderichs (2012) [29]	South Africa [2008–2010]	Routine—facility	Child health (hearing screening)	a. Extra services by immunization staff	EPI staff administered a hearing screening test; no non-integration comparison.	Not applicable/no relevant outcomes compared: feasibility study only.	**Not measured**	Proportion of infants that underwent hearing screening. No comparator.
Jac-Okereke (2020) [30]	Nigeria [ND]	Routine—facility	Child health (vision screening)	c. Co-location	Infants attending immunization clinics randomly selected for vision screening.	Not applicable/no relevant outcomes compared: descriptive study only.	**Not measured**	**Not measured**
Integration into routine immunization—family planning (FP) advice and services								
Balasubramaniam (2018) [31]	India [2012]	Routine—outreach	FP	b. Extra information and/or counseling by immunization staff	Providers applied a screening tool to assess for FP; both intervention and control areas updated in post-partum FP.	Mid-point/end-line; two groups [integration group = use of FP screening tool in intervention areas]	**DTP3 coverage (similar increases in intervention and control)**	**Percentage of women receiving FP services (no change in intervention, control decreased).** **FP methods (increased in intervention, but no change in control)**
Cooper (2015) [32]	Liberia [2012]	Routine—facility	FP	b. Extra information and/or counseling by immunization staff c. Co-location	Pilot of FP counseling by immunization staff at routine immunization visits and same-day referral to a co-located FP service.	Pre/post; two groups [integration group = pilot facilities comparator non-pilot facilities and pre-intervention data]	**-Provision of DTP1 and DTP3 (increase from pre to post and higher in pilot vs non-pilot in one site, but no differences in other sites)** -DTP1—3 drop-out rate (increased from pre to post and higher in pilot facilities compared to non-pilot at both sites).	**-New contraceptive users (increased from pre- to post-intervention in both sites)**
Cooper (2020) [33]	Malawi [2016–2017]	Routine—mixed (facility and outreach)	FP	a. Extra services by immunization staff c. Co-location e. Extra information and/or counseling by non-immunization staff	Same-day family planning services offered by nurses and health surveillance assistants to mothers seeking routine immunization services; screening of vaccination status for infants of family planning clients.	Pre/post; one group	**-Number of DTP1 doses (no change)** **-Number of DTP3 doses (increase from pre to post)**	**Number of women accessing FP services (increase from pre to post)**
Dulli (2016) [34]	Rwanda [2010–2011]	Routine—facility	FP	b. Extra information and/or counseling by immunization staff c. Co-location	Immunization staff provided information on FP and referral for counseling to co-located services. Compared to health facilities not using integrated approach.	Pre/post; two groups [integration group = health facilities implementing integrated family planning and immunization services]	**-Monthly trends in measles vaccinations (similar in integration and non-integration health facilities)** -Monthly trends in DTP1, 2, and 3 collected but not reported	**Proportion of women using a modern contraceptive method (higher in integration health facilities compared to non-integration group)**
Erhardt-Ohren (2020) [35]	Benin [2016–2017]	Routine—facility	FP	b. Extra information and/or counseling by immunization staff	Immunization staff provided information on FP and referral for FP counseling.	Not applicable/no relevant outcomes compared: qualitative process evaluation.	**Not measured**	**Not measured**
Nelson (2019) [36]	Liberia [2016–2017]	Routine—facility	FP	b. Extra information and/or counseling by immunization staff c. Co-location e. Immunization counselling by non-immunization staff	Immunization staff provided information on FP and referral for counseling to co-located services. Compared to matched facilities not using integration.	Pre/post; two groups [integration group = health facilities implementing integrated family planning and immunization services]	**-DTP1–3 drop-out rates (similar in integration and non-integration health facilities)**	**-FP referral (increased over time, higher in integration than non-integration)** **-Contraceptive acceptance (higher in integration than non-integration facilities)**
Vance (2014) [37]	Ghana and Zambia [2009–2010]	Routine—facility	FP	b. Extra information and/or counseling by immunization staff c. Co-location	Immunization staff provided information on FP and referral for services at nine-month visit at intervention health facilities. Outcomes compared to non-intervention (control) facilities.	Pre/post; two groups [integration group = health facilities where vaccinators were trained to provide FP messages and referrals to women presenting for child immunization services]	**Not measured**	**-Non-condom contraceptive use (no changes in intervention compared to control)** **-Women’s knowledge of LAM (no differences)** **-Referrals for FP services (non-significant changes across groups)**
Yugbare Belemsaga (2018) [38,39,40]	Burkina Faso [2013–2015]	Routine—facility	FP	a. Extra services by immunization staff b. Extra information and/or counseling by immunization staff c. Co-location	Provision of maternal-infant post-partum care checks and FP counseling at same time as immunization visits, by co-located staff.	Pre/post; one group	**-Monthly number of doses of DTP administered (no change over time)**	**-Uptake of post-partum care at immunization visits (no pre–post changes overall, some increase during the early post-natal period)**

^a^ the last name of the first author and the publication year; studies are ordered by linked maternal and child health service, and then alphabetically by author. Notes: DTP = diphtheria, pertussis, and tetanus vaccine; EPI = expanded program on of immunizations; FP = family planning; LAM = lactational amenorrhea method.

**Table 3:** Characteristics of included studies (*n* = 13) on integration of human immunodeficiency virus (HIV)prevention, testing, and treatment or malaria into routine immunizations, systematic review update 2011–2020.

Study ^a^	Country (Study Year(s))	Immunization Delivery Platform	Linked Maternal and Child Health Service(s)	Integration Strategy(ies)	Description of Integration Strategy	Study Design: Outcome Comparison	Immunization Outcomes Measured (Primary Outcome in Bold)	Linked Health Service Outcomes Measured (Primary Outcome in Bold)
Integration into routine immunization—HIV services								
Dube 2012 [41]	Malawi [2008–2010]	Routine—facility	HIV	c. Co-location	Testing, counseling, and treatment for EID of HIV offered at 6-week EPI visit.	Not applicable/no relevant outcomes compared: feasibility study to describe completion of EID process during routine EPI visits.	**Not measured**	-Measured, but no non-integration comparator -71.6% of HEIs had early diagnosis, 87.3% of mothers informed of infants’ results, ART started in 58% of infants with confirmed infection.
Goodson (2013) and Wallace (2014) [42,43]	Tanzania [2009–2010]	Routine—facility	HIV	a. Extra services by immunization staff b. Extra information and/or counseling by immunization staff c. Co-location	EPI staff identified HEIs offered testing, counseling and care in selected sites. Compared to remaining sites in district (control).	For immunization outcome: pre/post; two groups [integration group] For linked service: not applicable/no relevant outcomes compared—feasibility study only.	**-Monthly vaccine doses [OPV1, OPV3, Penta1, Penta3, Measles] (results differed by urban/rural and by antigen; overall either no change or a decrease in monthly doses)**	**-Measured, but no non-integration comparator** -HIV service uptake increased in all sites
McCollum (2012) [44]	Malawi [2011]	Routine—facility	HIV	b. Extra information and/or counseling by immunization staff c. Co-location	Testing and counseling for EID of HIV offered at EPI clinic. Compared to EID offered at pediatric clinic.	Post only; two groups [integration group = infants attending EPI clinic where EID service was co-located]	**Not measured**	**-Proportion of infants receiving provider initiated HIV testing and counseling for EID (higher in EPI than pediatric clinic)**
Odafe (2020) [45]	Nigeria [2016–2017]	Routine—facility	HIV	c. Co-location	Targeted HIV testing of infants in immunization clinics compared to in TB clinics, inpatient wards, outpatient clinics, and through a family registry system.	Post only; five platforms/groups [integration group = immunization clinics]	**Not measured**	**-Proportion of all tests by platform (testing at immunization clinics low [0.9%] compared to others: pediatric outpatients [49.7%], family registry [38.0%], pediatric inpatient [10.5%], or tuberculosis [0.8%] clinics)**
Ong’ech (2012) [46]	Kenya [2008–2010]	Routine—facility	HIV	c. Co-location	Services for HEIs (infant feeding support, prophylactic medicines, and HIV diagnosis) offered at MCH clinics with EPI services co-located compared to specialized HIV clinic.	Post only; two groups [integration group = infants seeking care at MCH clinic offering EPI and HEI services]	**-Measles vaccine coverage at 9 months (higher in integrated MCH clinic than specialized HIV clinic)** **-Completion of vaccinations by 12 months (higher in integrated MCH clinic than specialized HIV clinic)**	**-Odds of attending scheduled post-natal visits (higher in integrated MCH clinic than specialized HIV clinic)** **-Proportion of infants attending follow-up (higher in integrated MCH clinic than specialized HIV clinic)**
Tejiokem (2011) [47]	Cameroon [2007–2009]	Routine—facility	HIV	c. Co-location	Testing, counseling, and treatment for EID of HIV offered at 6-, 10-, and 14-week EPI visit and linkage to HAART.	Not applicable/no relevant outcomes compared: feasibility study only.	**Not measured**	-Measured, but no non-integration comparator -Completeness of follow-up of HEIs (83.9% completed)
Wang (2015) [48]	Zambia [2012–2014]	Routine—facility	HIV	a. Extra services by immunization staff b. Extra information and/or counseling by immunization staff c. Co-location	EPI and co-located staff, with commodity support, offered HIV testing and counseling.	Pre/post; three groups [control, simple intervention = re-supply of HIV commodities, integration group = integration of HIV testing with routine immunization]	**-Monthly DTP1 doses (similar change in both intervention arms and matched control sites)** -Qualitative finding: HIV services did not deter families from immunization.	**-Monthly HIV tests administered (higher in integration compared to simple intervention and control)**
Integration into routine immunization—malaria prevention								
Bojang (2011) [49]	Gambia [2006]	Routine—outreach	Malaria	a. Extra services by immunization staff	IPTc during outreach by community-based VHWs) compared to outreach by reproductive and child health trekking teams (EPI staff).	Post only; two groups [integration group = RCH teams (EPI staff) delivery]	**% children ≥ 12 months who were fully vaccinated (lower in RCH teams compared to non-integrated VHW delivery)**	**-Coverage of three courses of IPTc (lower in RCH teams delivery compared to non-integrated VHW)** -Cost of delivery at least first dose of IPTc (more expensive to deliver by RCH teams compared to VHWs)
Dicko (2011) [50,51]	Mali [2006–2007]	Routine—facility	Malaria	a. Extra services by immunization staff	IPTi services and counseling by staff at EPI clinics in intervention sites. Compared to sites not offering IPTi.	Pre/post; two groups [integration group = randomized areas where IPTi was delivered with routine vaccinations]	**Coverage of fully vaccinated children 9–23 months [BCG, 3 doses of DTP, 3 doses of polio, measles, and yellow fever vaccines] (increase in coverage in intervention compared to control and baseline)**	**-Coverage of IPTi (higher in intervention compared to control)** -Coverage of vitamin A (higher in intervention compared to control) -ITN use (similar in intervention compared to control)
Patouillard (2011) [52]	Ghana [2006]	Routine—outreach	Malaria	a. Extra services by immunization staff b. Extra information and/or counseling by immunization staff	IPTc delivery by VHWs compared to facility-based staff (at outpatient departments or EPI outreach clinics).	Post only; two groups [integration group = facility-based delivery]	**Not measured**	**-Coverage of IPTc (similar in VHW delivery and facility-based delivery)** -Cost of delivering full course of IPTc (more expensive to deliver by EPI nurses compared to VHWs)
Scates 2020 [53]	Ghana, Mali, Tanzania [2015–2016]	Routine—mixed (facility in Ghana, Tanzania, and Mali; community and facility in Zanzibar)	Malaria	a. Extra services by immunization staff	Continuous distribution of insecticide-treated bednets (ITNs) by immunization staff through ANC, EPI, schools, and mass distribution campaign.	Not applicable/no relevant outcomes compared: descriptive study of costs associated with various ITN continuous distribution delivery platforms.	**Not measured**	**Not measured**
Schellenberg (2011) and Willey (2011) [54,55]	Tanzania [2005–2007]	Routine—facility	Malaria	a. Extra services by immunization staff	IPTi services and counseling by staff at EPI clinics in intervention sites. Compared to no IPTi control sites.	Pre/post for infant survival and post only for other outcomes; two groups [integration group = randomized areas where IPTi was delivered with routine vaccinations compared to no IPTi control]	**-DTP3 and measles vaccination coverage (similar in IPTi vs no IPTi groups)**	**-Coverage of three doses of IPTi (higher in intervention compared to control)** -Infant mortality (decrease over time, but no difference between intervention and control groups)
Theiss-Nyland (2016b) and (2017) [56,57]	Kenya, Malawi, Mali, Rwanda [2014]	Routine—mixed	Malaria	a. Extra services by immunization staff	Continuous distribution of LLINs through ANC and EPI	Not applicable/no relevant outcomes compared: feasibility study of continuous distribution.	**Not measured**	**Not measured**

^a^ the last name of the first author and the publication year; studies are ordered by linked maternal and child health service, and then alphabetically by author. Notes: ANC = antenatal care; ART = antiretroviral therapy; BCG = bacille Calmette–Guerin vaccine; DTP = diphtheria, pertussis, and tetanus vaccine; EID = early infant diagnosis; EPI = expanded program of immunizations; HEI = HIV-exposed infant; HIV = human immunodeficiency virus; IPTc/i = intermittent preventive treatment for malaria during infancy/in children; ITN = insecticide-treated bednet; LLIN = long-lasting insecticidal nets; MCH = maternal and child health; OPV = oral polio vaccine;Penta = pentavalent vaccine; VHW = village health worker.

**Table 4:** Characteristics of included studies (*n* = 7) on the integration of primary healthcare services, nutrition, water, sanitation, and hygiene (WASH), or a combination of services with routine immunizations, systematic review update 2011–2020.

Study ^a^	Country (Study Year(s))	Immunization Delivery Platform	Linked Maternal and Child Health Service(s)	Integration Strategy	Description of Integration Strategy	Study Design: Outcome Comparison	Immunization Outcomes Measured (Primary Outcome in Bold)	Linked Health Service Outcomes Measured (Primary Outcome in Bold)
Integration into routine immunization—maternal depression screening								
Bakare (2014) and (2017) [58,59]	Nigeria [2012]	Routine—facility	PHC—maternal depression	c. Co-location	Depression screening service co-located with EPI clinic visits.	Not applicable/no relevant outcomes compared: feasibility study only.	**Not measured**	**Not measured**
Integration into routine immunization—nutrition activities								
Monterrosa (2013) [60]	Mexico [2011]	Routine—mixed	Nutrition	b. Extra information and/or counseling by immunization staff	EPI nurses and radio communications delivered scripted nutrition counseling messages. Behavior change among women with children 6–24 months compared between with and without messages.	Pre/post; two groups [integration group = sites receiving nutrition communications from EPI nurses and via radio]	**Not measured**	**-Frequency of breast feeding (higher in integration group compared to sites not receiving nutrition communications)** -Self-reported feeding (mixed results but some healthier consumption in integration group)
Oladeji (2019) [61]	South Sudan [2017]	Routine—mixed (nutrition treatment centers and community outreach)	Nutrition	c. Co-location e. Extra information and/or counseling by non-immunization staff	Immunization services offered to children visiting nutrition treatment clinics and nutrition community outreach. Nutrition volunteers provide messages on immunization, screen vaccination status and track children. Compared to immunization atPHCs.	Post only; two groups [integration group = immunization delivery at nutrition treatment clinics and community outreach]	-Proportion of children vaccinated in nutrition treatment clinics compared to standard PHC clinics [BCG, OPV 0–3, Penta 1–3, IPV, and measles] (data insufficient for comparison) **-Drop-out Penta1 to Penta3 (lower drop-out in nutrition treatment clinics compared to PHCs)**	**Not measured**
Integration into routine immunization—water, sanitation, and hygiene interventions (WASH)								
Briere (2012) and (Ryman) 2012 [62,63]	Kenya [2009]	Routine—facility	WASH	a. Extra services by immunization staff b. Extra information and/or counseling by immunization staff c. Co-location	During routine immunization visits, health workers offered water treatment, soap and related education to caregivers children aged <1 yr old.	Pre/post; two groups [integration group = district randomly selected to integrate WASH interventions at routine immunization visits]	**-Percent of children 2–20 months who received all vaccine doses [penta, OPV, and measles] (higher in intervention compared to control)** **-Percent of children 2–20 months who received all age-appropriate vaccines (higher in intervention compared to control)**	**-Awareness of water treatment options (increase in both intervention and control from pre to post)** **-Uptake of water treatment (significant pre–post increase in intervention, not control)** **-Percentage of households with soap (pre–post increase in intervention, not control)**
Integration into routine immunization—multiple primary healthcare and disease control interventions								
Bawa (2019) [20,21]	Nigeria [2014–2015]	Routine—mobile	Malaria, PHC, Nutrition, WASH	c. Co-location	Package of MCH services (antenatal care, malaria prevention, iron folate, deworming, child health, WASH, vitamin A, nutrition screening, and routine immunizations) delivered to women and children during mobile outreach visits.	Pre/post; one group	**-Coverage of measles vaccination (increased from pre to post)**	**-Provision of multiple MCH interventions (increase from pre to post)**
Edmond (2020) [22]	Afghanistan [2013]	Routine—mobile	PHC, Nutrition	c. Co-location	Integrated outreach services for ANC, PNC, and immunization by mobile health teams (MHTs).	Post only; two groups [integration group = recent MHT integrated outreach delivery, comparator = areas without recent visits]	**-Measles first dose coverage (higher in areas with recent MHT integrated outreach compared to non-recent)** **-Penta 3 coverage (higher in areas with recent MHT integrated outreach, but not statistically significant)**	**-Coverage of at least one ANC visit (higher in areas with recent MHT integrated outreach)** **-Coverage of at least one PNC visit (no difference)** **-Coverage of facility-based delivery (no difference)** **-Coverage of at least one IMCI visit (higher in areas with recent MHT integrated outreach)**
Hodges (2015) [24]	Sierra Leone [2012–2013]	Routine—facility	Family planning, Nutrition	c. Co-location e. Extra information and/or counseling by non-immunization staff	Extra co-located staff provided vit A and either IYCF counseling or IYCF and FP services, alongside routine and catch-up vaccination, during visits from 6 months of age onwards. Extra staff also gave information on catch-up vaccination.	Post only; three groups [integration group 1 = revised vaccination card with vit A + IYCF + FP counseling, integration group 2 revised vaccination card with vit A, control group = revised vaccination card with vit A]	**-Proportion of fully vaccinated children (similar in all groups)** **-Proportion of infants receiving catch-up vaccination [OPV 2 or 3, penta and/or PCV] (higher in both integration groups compared to control)**	**-Coverage of vit A supplement (higher in both integration groups compared to control)** **-Provision of FP counseling and commodities (higher in both integration groups compared to control)**

^a^ the last name of the first author and the publication year; studies are ordered by linked maternal and child health service, and then alphabetically by author. Notes: ANC = antenatal care; BCG = bacille Calmette–Guerin vaccine; EPI = expanded program of immunizations; FP = family planning; IMCI = integrated management of childhood illnesses; IPV = inactivated polio vaccine; IYCF = infant and young child feeding; MCH = maternal and child health;; OPV = oral polio vaccine; PHC = primary healthcare; PCV = pneumococcal conjugate vaccine; Penta = pentavalent; PNC = prenatal care; vit A = vitamin A supplementation; WASH = water, sanitation, and hygiene.

### 3.3. Integration Strategies

Almost half (44%) of the included studies reported the use of multiple strategies for integrated service delivery (Table 4). The most frequently described strategies were the co-location of services (*n* = 23 studies, 68%), the provision of extra services by immunization staff (*n* = 14, 41%), the provision of extra information and/or counseling by immunization staff (*n* = 14, 41%), and the provision of extra information and/or counseling by non-immunization staff (*n* = 4, 12%). No studies reported on vaccination services provided by staff whose job description did not usually include vaccination.

In general, there was no clear pattern in the types of strategy used to integrate particular linked MCH services (Table 1, Table 2, Table 3 and Table 4). However, it was noted that the provision of ‘extra services by immunization staff’ was one of the integration strategies described in 88% (7 of 8) of malaria studies [25,26,49,50,51,52,53,54,55,56,57] and the provision of ‘extra information and/or counselling by immunization staff’ was one of the strategies described in 78% (7 of 9) of family planning studies [31,32,34,35,36,37,38,39,40].

### 3.4. Effect of Integration on Outcomes for Immunization and Linked Service

Most of the included studies (*n* = 25, 74%) reported at least one immunization (*n* = 20) and/or linked health service outcome (*n* = 21) that could be compared across groups with and without the implementation of integrated service delivery (Table 5). Data on immunization outcomes included vaccination coverage for one or more antigens or the proportion of children who were fully vaccinated (*n* = 11, 55%), the number of doses for one or more antigen (*n* = 7, 35%), and drop-out rates (*n* = 2, 10%). For linked MCH services, the reported outcomes either described the coverage of the service (*n* = 14, 67%) or the number of participants accessing/using the service (*n* = 7, 33%). Overall, the effect of integration was beneficial (mixed positive or positive) for most (76%) studies reporting linked MCH service outcomes and nearly half (55%) of studies reporting immunization outcomes.

For most studies that reported both immunization service and linked health service outcomes (*n* = 16), the relative boost in coverage or uptake obtained for the linked MCH service was generally the same or better than the effect on the immunization outcome. No clear relationship was observed between the integration strategy and the effect when grouped by immunization delivery platform and linked health service (Table 5).

Among studies that integrated service delivery during immunization campaigns (*n* = 3), groups receiving integrated services had significantly higher vaccination coverage for measles [25,26] and oral polio vaccine (OPV) [23,28], and significantly higher long-lasting insecticide-treated (ITN) bednet coverage [25,26], compared to non-integrated service delivery groups (Table 5).

For studies of the integration of family planning services with routine immunization service delivery (*n* = 7), the effect on vaccination coverage was static. The exception was the work of Cooper, 2020 [33], which showed a mixed positive effect based on the static coverage of DTP1 but elevated coverage of DTP3. Across the same studies [31,32,33,34,36,37,38,39,40], the effect on family planning service outcomes ranged from static to positive (Table 5).

The effect on outcomes was more variable in the seven studies integrating HIV services with routine immunization service delivery [42,43,44,45,46,48]. The three studies reporting immunization outcomes showed mixed negative [42,43], static (48), or positive [46] effects on either vaccination coverage or the number of doses administered. The four studies reporting linked health service outcomes indicated mostly positive effects [44,46,48], but one negative (45) effect, on outcomes related to early infant diagnosis of HIV (Table 5).

Among the six studies integrating malaria interventions with routine immunization service delivery [49,50,51,52,54,55], the direction of the effect on outcomes varied. However, within studies, there was some consistency in the outcome effects on both the immunization and linked MCH services. Bojang (2011) [49] reported the negative effects of integration on both fully vaccinated coverage and the coverage of intermittent preventative treatment for malaria in children (IPTc) and Dicko (2011) [50,51] reported positive effects on both fully vaccinated coverage and the coverage of IPT during infancy (IPTi). Schellenberg (2011) and Wiley (2011) [54,55] reported a static effect on DTP3 and measles vaccination coverage, but a positive effect on IPTi. Patouillard (2011) [52] did not measure immunization outcomes and reported a static effect of integration on IPTc (Table 5).

Two studies of the integration of nutrition services with routine immunization service delivery only reported outcomes related to either immunization or linked health services. Monterrosa (2013) [60] reported the positive effect of integration on the frequency of breastfeeding and Oladeji (2019) [61] reported a positive improvement in from Penta 1 to Penta 3 drop-out rates in the integrated group (Table 5).

Two studies focused on the integration of WASH services with immunization services. Briere (2012) and Ryman 2012 [62,63] both reported statistically significant increases in the proportion of fully vaccinated children, and increases in WASH outcomes such as awareness and the uptake of water treatment options in the integrated service delivery group compared to the non-integrated delivery group (Table 5).

For the four studies integrating multiple services with routine immunization service delivery [20,21,22,23,24], the effects of integration on immunization outcomes and linked service outcomes were either mixed positive or positive (Table 5).

### 3.5. Implementation Considerations

The processes, enablers, and barriers affecting service integration, as described by the included studies, are shown in Table 6.

In relation to policy and governance, the key themes that emerged were the consistency of national government support and policies for integration [25,26,28,34,37,48]; the pursuit of efficiency in the delivery of health services by governments and donors, in particular for campaigns [23,33]; and the expansion of policies to minimize the financial burden of integration within primary healthcare services [38,39,40] (Table 6).

Several studies discussed the re-design of services in a way best suited to promote integration. One frequently mentioned consideration was the need for the co-location in place and time of additional staff, particularly if the linked service was time-consuming or needed specialized skills [32,33,36,37,61]. Additionally, studies also noted that integrated approaches should consider the compatibility of cadence for services, target groups, and types of services [38,39,40,44]; McCollum (2012) [44] noted that the integration of HIV preventative services with immunization may be more beneficial than treatment due to the better prognoses obtained when initiating care as early as possible. Studies also reported that the process of integration should not compromise the uptake or quality of services [6,41,42]. Integration was also sometimes viewed as an opportunity to provide services, especially with the help of community health workers, to areas or communities that are hard to reach, thus expanding the reach of services [20,21,23,25,26,61] (Table 6).

Most studies mentioned one or more considerations relating to human resources for health that affect the success of integrated service delivery. The training needs highlighted included the benefit of harmonized combined training for vaccinators and linked health services providers [20,21,23,24,27,29,32,34,36,37,38,39,40,46,49,50,51,54,55], additional training on communications to improve community understanding and the uptake of integrated activities [23,32,33,34,36,56,61,62,63], and training focused on integration processes and tools [32,33,34,36,45]. The management of staff workloads and the time required for additional tasks were also major concerns reported in many studies [22,29,36,37,38,39,40,49,50,51,52,53,56,57,60,62,63]. There was recognition that integration may require additional staff with specific skillsets [20,21,22,28,35,36,37,38,39,40,44,49,52]. Other considerations mentioned were the importance of involving community-based health workers in efforts to support integrated service delivery [20,21,24,33,44,45,46,48,49,52,61,62,63], improved motivation, the empowerment of healthcare staff to provide expanded care, which healthcare workers generally perceived as better in terms of quality and responsiveness [32,33,36,48,50,51], and ensuring that staff involved in integration were willing to collaborate in order to provide the additional services [37,38,39,40] (Table 6). In terms of management, logistics, and costs, the consistent availability of supplies and commodities, particularly for the linked service, and coordinated logistics were mentioned frequently, with some studies noting that stockouts could be a deterrent for clients returning for care or services [20,21,30,32,35,36,46,48,49,50,51,52,56,57,62,63]. Particularly for more sensitive linked services, such as HIV or family planning, studies noted that it was important to ensure that infrastructure for service delivery was supportive and appropriate regarding considerations such as privacy needs [29,32,35,36,42,43]. Additionally, although some studies suggested that improving efficiencies could reduce the costs of integrated service delivery [27,34,38,39,40,52,62,63], one study noted that integration involving professional staff may, in fact, increase costs [49] (Table 3). Studies noted the need for new systems for reporting and recording integrated service delivery. Other considerations included new tools, such as job aids to support staff in providing integrated care or other operational aspects of integration [20,21,31,32,33,34,36,56,57]. There were also reports of the need to account for the additional workload in expanded information and reporting systems [29,31,32,33,34,36,37,38,39,40,45,57], including home-based records, and ensure adequate time is taken to record and report additional services [36,56,57] (Table 6).

Many studies reported the importance of addressing community and client considerations during service re-design; specifically, there was a need for some consideration of how integrated services would be perceived and the degree to which they would be seen as more responsive to clients’ needs [23,24,27,28,31,36,49,50,51,57,61,62,63]. The need for clear communications through multiple channels was reported in some studies [25,26,33,38,39,40]. In scenarios where linked services were potentially sensitive, such as for HIV diagnosis or care, integration efforts needed to address potential stigma [23,32,33,34,35,36,41,42,43,46,58,59], and for some, like family planning, they needed consider how to account for clients’ preferences in terms of involving a spouse in decision-making [32,35,41]. Clients, like providers, were concerned that integrating services should not unreasonably increase the time or costs of care seeking [22,38,39,40,41,42,43,46,62,63]. Many studies did consider the degree to which more integrated services could address common constraints on care seeking, and this was reported as an important motivator for pursuing health service integration [22,27,28,32,33,34,35,36,38,39,40,41,42,43,44,46,61,62,63] (Table 6).

## 4. Discussion

In this systematic review update of the integration of MCH services with childhood immunization services, we synthesized evidence from 34 relevant studies, which were identified between 2011 and 2020, following the publication of the last review on this topic in 2012 [6]. In comparison to previous reviews [4,6], there were fewer articles included reporting on the integration of service delivery within immunization campaigns. With more recent global strategies calling for a shift towards integrated campaigns beyond the co-delivery of multiple vaccine antigens [64], evidence from integrated service delivery in campaigns will be important for informing these approaches. Although a similar range of linked services was observed in this review as in the previous reviews [4,6], the current review included a larger proportion of family planning studies. Additionally, despite recent global initiatives calling for integrated service delivery within primary healthcare structures [1,2,8], most included studies focused on malaria, family planning, and HIV, suggesting that the implementation of integrated service delivery may be driven by specific programs or funded initiatives rather than by a push towards achieving universal health coverage. The considerable heterogeneity of study designs reflects the challenges of conducting real-world implementation research into modifications to service delivery, especially in low-resource settings.

Our review assessed the effect of integrated service delivery on outcomes for immunization and/or linked health services. Among 16 included studies where both outcomes were reported, the effect of integration on the linked health service was generally equal to or showed greater benefit than the effect on immunization; additionally, the effect on immunization was only negative in one study where both outcomes were reported. This result supports previous observations that the coverage of other health services could be expanded through integrated service delivery with immunization programs. This was described, for example, in reviews from 2015 relating to interventions for HIV [65] and family planning [66]. In these, and in our synthesis, the extent of benefit was likely to depend on the strength of the immunization platform prior to integration [6] and on the successful management of implementation considerations. Positive outcomes were not universal: we found two studies in which integrated service delivery had poorer outcomes for the linked service when compared to alternatives for both HIV [45] and malaria [49]; immunization outcomes were not reported in either of these studies. For both studies, there were different processes contributing to the lack of benefit to linked health services from integration. In the HIV study [45], immunization clinics yielded the lowest percentage of eligible children for pediatric HIV testing in comparison with other points of service, possibly due to the lower overlap of the target age range for immunization clinics and pediatric HIV testing (0–14 years). In the malaria study [49], the coverage of IPTc was higher when delivered by village health workers compared to monthly delivery by reproductive and child health trekking teams, which included EPI staff. This may have been due to the convenience of community-based antimalarial delivery, which is available any day of the month rather than on scheduled EPI delivery days. In one study, which integrated family planning with immunization [37] and did not see increased contraceptive uptake, a lack of fidelity in implementation was assessed as the likely cause. Community perceptions appear to play an important role, with caution seen when HIV service referrals are integrated with immunization visits [43], and enthusiasm seen when immunization is linked to malaria control [51].

The previous reviews [4,6] highlighted challenges with integration including, logistical difficulties, concerns with harming existing services, target age group differences, and pre-existing low immunization coverage. Key lessons learned for service delivery integration included the importance of establishing intervention compatibility, considering the current immunization program’s strength when choosing to integrate, and ensuring strong planning for integration. In this review, almost all studies noted the critical importance of the details of implementation in determining whether an attempt to integrate services proves beneficial. The considerations summarized in Table 3 provide a framework that can be drawn on by planners wishing to promote greater service integration. Our synthesis suggests that three key areas determine how easy it is to integrate services, and thus contribute to improved service delivery: firstly, whether there is a good alignment of the new linked services’ target population and the need for skills and supplies with the capacities of the service platform already in use for immunization; secondly, whether investments are in place to adequately support staff in terms of the guidance, training, time, supplies, and workforce needed to deliver the expanded package of care; and thirdly, whether clients’ expectations (for example privacy or timely care) are met and community perceptions are well understood, which can be achieved through formative assessment if necessary. These areas were not demonstrated in all included studies, and relatively fewer studies fully accounted for the client perspective; therefore, this represents our synthesis of the factors that correlated with beneficial outcomes.

In this review, we observed a range of integration processes, such as child health screening; the provision of referrals, counseling, or testing; and the distribution of commodities or medicines. We broadly classified these into types of strategies, such as the provision of extra services or information by immunization or non-immunization staff, and the co-location or timing of service delivery. Most studies reported the use of a combination of these strategies in the integration process, with co-location and the provision of extra services by immunization staff being the most common. The feasibility of the integration strategy depends on the complexity of the linked health service and the EPI platform used for delivery; for instance, easy-to-administer commodities such as ITNs or medicines such as de-worming treatment may be more conducive to extra service provision by immunization staff, either during immunization campaigns or at routine immunization contact points, but more time-intensive and potentially sensitive processes such as counseling for HIV testing and treatment may be better suited to delivery during routine immunization services [5]. Our evidence also suggests that the integration of complex tasks, especially those involving counseling or curative care, may be better handled by the co-location of additional staff at the same time and place as immunization services, rather than via the addition of these tasks to the work of vaccinators.

To enable a focus on integration outcomes, this review was limited to studies reporting on intentional efforts to implement and measure an explicit change to service delivery with the prime purpose of integrating other services with childhood immunization programs. We could not include broader approaches such as the integrated management of childhood illnesses, for which integration is one approach amongst several, as part of primary healthcare-strengthening initiatives; these may also provide valuable examples. Additionally, given this review’s focus on integrated service delivery at immunization program contact points, we excluded studies reporting on missed opportunities for vaccination and integrated approaches to community health education, which are other key focus areas for integration with potential benefits to immunization coverage. Additionally, given the wide range of study designs and the heterogeneity of outcomes reported, we were unable to conduct a quantitative synthesis of effect measures.

Our review identified new articles published since the previous review on this topic [6], but several evidence gaps remain. First, there were few studies from LMICs outside of Africa; additional studies from a wider range of LMICs are needed, particularly from Asian and Pacific nations, given their large populations and/or the knowledge gaps. Second, many studies reporting on integrated campaigns did not include a non-integration comparator, thus limiting our ability to evaluate the effect of integrated service delivery; there is a pressing need to expand the documentation of integrated health campaigns, especially regarding their cost-effectiveness [64,67]. Third, although the cost-saving benefits of integrated services in general are sometimes cited [1], there were few studies reporting on the cost of integrated service delivery compared to the use of non-integrated approaches. Fourth, evidence gaps persist surrounding the role of client preferences for integrated service delivery, including which services to integrate, at what contact points, and how, in the design of integrated approaches. Relatively few of the studies we reviewed included community members’ preferences in the design of integrated services. Finally, future implementation research should focus on integrated service delivery in the context of new vaccine introduction, in particular for older age groups beyond the first two years of life. More recently introduced vaccines a in adolescence, such as those against the human papillomavirus (HPV), present a new range of opportunities for integrating other services; these vary considerably by service delivery platform, with different options for integration during school-based vaccination to those available from facility- or community-based services [68].

## 5. Conclusions

Approaches to integrating MCH services with immunization programs have the potential to expand access to and the utilization of linked services and, in some cases, improve immunization coverage. The strategies and processes for integration varied across studies, and were typically determined by the nature of the linked health service. The details of service design and implementation were found to be critical determinants of the success of integration. Integrated services continue to play an important role in achieving more client-centered services in primary healthcare and progress towards IA2030. Future implementation research should expand the focus of integrated service delivery by taking a life-course approach that examines opportunities for expanded care during immunization services offered at broader age ranges.

## Figures and Tables

**Table 5:** A qualitative summary of the effect of integrated service delivery on immunization and linked service outcomes (*n* = 25 *) by immunization delivery platform, linked health service, and a combination of integration strategies, systematic review update 2011–2020.

				Qualitative Summary ^1^ of the Effect of Integrated Service Delivery											
				Immunization Outcome ^2,3^						Linked MCH Service Outcome ^2,3^					
Study	Country	EPI Platform	Linked Health Service(s)	NOT MEASURED	NEG	M-NEG	STATIC	M-POS	POS	NOT MEASURED	NEG	M-NEG	STATIC	M-POS	POS
Goodson 2012 & Kulkarni 2010 [25,26]	Madagascar	Campaign—mixed	Malaria						**A C**						**A C**
Birukila 2017 [23]	Nigeria	Campaign—mixed	PHC						**C**	**C**					
Habib 2017 [26]	Pakistan	Campaign—mixed	Nutrition, PHC						**B C**	**B C**					
Balasubramaniam 2018 [31]	India	Routine—outreach	FP				**B**							**B**	
Cooper 2015 [32]	Liberia	Routine—facility	FP				**B C**								**B C**
Dulli 2016 [34]	Rwanda	Routine—facility	FP				**B C**								**B C**
Vance 2014 [37]	Ghana, Zambia	Routine—facility	FP	**B C**									**B C**		
Cooper 2020 [33]	Malawi	Routine- mixed	FP					**A C E**							**A C E**
Nelson 2019 [36]	Liberia	Routine—facility	FP				**B C E**								**B C E**
Yugbare Belemsaga 2018 [38]	Burkina Faso	Routine—facility	FP				**A B C**						**A B C**		
Odafe 2020 [45]	Nigeria	Routine—facility	HIV	**C**							**C**				
Ong’ech 2012 [46]	Kenya	Routine—facility	HIV						**C**						**C**
McCollum 2012 [44]	Malawi	Routine—facility	HIV	**B C**											**B C**
Goodson 2013 &Wallace 2014 [42,43]	Tanzania	Routine—facility	HIV			**A B C**				**A B C**					
Wang 2015 [48]	Zambia	Routine—facility	HIV				**A B C**								**A B C**
Bojang 2011 [49]	Gambia	Routine—outreach	Malaria		**A**						**A**				
Dicko 2011 [50]	Mali	Routine—facility	Malaria						**A**						**A**
Schellenberg 2011 & Willey 2011 [54,55]	Tanzania	Routine—facility	Malaria				**A**								**A**
Patouillard 2011 [52]	Ghana	Routine—outreach	Malaria	**A B**									**A B**		
Monterrosa 2013 [60]	Mexico	Routine—mixed	Nutrition	**B**											**B**
Oladeji 2019 [61]	South Sudan	Routine—mixed	Nutrition						**C E**	**C E**					
Briere 2012& Ryman 2012 [62,63]	Kenya	Routine—facility	WASH						**A B C**						**A B C**
Bawa 2019 [21]	Nigeria	Routine—mobile	Malaria, PHC, Nutrition, WASH						**C**						**C**
Edmond 2020 [22]	Afghanistan	Routine—mobile	Nutrition, PHC						**C**					**C**	
Hodges 2015 [25]	Sierra Leone	Routine—facility	FP, Nutrition					**C E**							**C E**

Notes: EPI = expanded program of immunizations; HIV = human immunodeficiency virus; FP = family planning; MCH = maternal and child health; PHC = primary healthcare; WASH = water, sanitation, and hygiene. * studies that reported at least one immunization and/or linked health service outcome. ^1^ primary outcomes that were considered in this qualitative summary are designated in bold in Table 1. ^2^ a single qualitative summary was used to describe the direction of effect of integration for the primary outcome(s) for immunization and for the linked health service separately. The terms used were defined as follows: “not measured”—no outcome measured or no non-integration comparison group available; “negative”—lower coverage or service provision in the integration compared to the non-integration group; “mixed negative”—some outcomes were lower in the integration compared to the non-integration group but others remained static; “static”—no meaningful difference in coverage or service provision between integration and non-integration groups; “mixed positive”—some outcomes were higher in the integration compared to the non-integration group, but others remained static; or “positive”—higher coverage or services in the integration compared to the non-integration group. ^3^ integration strategies were categorized in one or more of the following ways: A. extra services provided by immunization staff (i.e., testing, treatment, referrals, etc.); B. extra information and/or counseling provided by immunization staff (i.e., one-on-one consultation, the provision of informational materials, etc.); C. the co-location of services (e.g., same timing or same physical location of immunization delivery and linked health service); D. vaccination provided by non-immunization staff; E. extra information and/or counseling provided by non-immunization staff (i.e., screening, health education, or immunization promotion, etc.).

**Table 6:** Implementation considerations reported for integrated service delivery of maternal and child health services with immunization programs, systematic review update 2011–2020.

Category	Processes, Enablers, and Barriers Affecting Service Integration	References
Policy and governance topics	Consistency of leadership support	[25,26,28,34,37,48]
	Governments and donors seeking efficiency amidst multiple priorities	[23,33]
	Reducing financial access barriers to the integration of other primary healthcare services with immunization	[23,33,38,39,40]
Design of integrated service delivery	Optimize co-location of additional staff, especially if extra tasks require more time (e.g., counseling) or skills	[32,33,36,37,61]
	Ensure good matches in terms of of cadence, target groups, and types of services (e.g., preventive rather than curative services may more amenable to integration with immunization)	[38,39,40,44]
	Ensure additional service requirements do not compromise uptake or quality	[41,42,43]
	Use integration as an opportunity to provide services to areas/populations that are difficult to reach	[20,21,23,25,26,61]
Human resources for health	Managing workload and time demands for extra tasks	[22,29,36,37,38,39,40,49,50,51,52,53,56,57,60,62,63]
	Additional and skilled staff may be needed (e.g., counseling)	[20,21,22,28,35,36,37,38,39,40,44,49,52]
	Motivation and empowerment of healthcare staff to provide expanded care, which healthcare workers generally perceive as better in quality and responsiveness	[32,33,36,48,50,51]
	Training that is (a) harmonized for vaccinators and for providers of linked health service; (b) includes client communications/mobilization; and (c) includes integration processes and tools	[20,21,23,24,27,29,32,33,34,36,37,38,39,40,45,46,49,50,51,54,55,56,61,62,63]
	Involving community-based health workers to support integrated service delivery	[20,21,24,33,44,45,46,48,49,52,61,62,63]
	Ensuring that staff are willing to collaborate in providing additional services	[37,38,39,40]
Management, logistics, and costs	Coordinated program planning across program managers and between staff	[32,33,44,48,49]
	Consistency of supplies (particularly for linked service) and coordinated logistics	[20,21,30,32,35,36,46,48,49,50,51,52,56,57,62,63]
	Efficiency may lower costs of integrated service	[27,34,38,39,40,52,62,63]
	Integrating professional staff may increase costs	[49]
	Ensuring infrastructure is supportive (e.g., privacy)	[29,32,35,36,42,43]
Operational tools, reporting and recording	Consolidation of information systems and reporting, including home-based records	[20,21,31,32,33,34,36,56,57]
	Ensuring time for documentation of extra services	[36,56,57]
	Job aids and other tools to support implementation of linked service and referral between programs	[29,31,32,33,34,36,37,38,39,40,45,57]
Community and client topics	Promote care seeking, with perceptions of integrated services as more responsive and client-focused	[23,24,27,28,31,36,49,50,51,57,61,62,63]
	Ensuring stigma/sensitivity does not hamper uptake	[23,32,33,34,35,36,41,42,43,46,58,59]
	Ensuring family time and travel costs do not increase	[22,38,39,40,41,42,43,46,62,63]
	Account for need to involve spouse in care decisions	[32,35,41]
	Ensuring integrated services address common barriers to uptake	[22,27,28,32,33,34,35,36,38,39,40,41,42,43,44,46,61,62,63]
	Ensuring multiple channels of communication on integrated health services	[25,26,33,38,39,40]

Note: this table outlines the implementation considerations extracted from studies, reporting contributors to the degree to which service integration affected outcomes for the linked service or immunization.

## Data Availability

The original contributions presented in this study are included in the article/Supplementary Materials. Further inquiries can be directed to the corresponding author(s).

## Supplementary Materials

The following supporting information can be downloaded at https://www.mdpi.com/article/10.3390/vaccines12121313/s1. Table S1: List of studies excluded on full-text review and reason for exclusion (*n* = 427).
