# Supplementary material for: Integrated Approaches for the Delivery of Maternal and Child Health Services with Childhood Immunization Programs in Low- and Middle-Income Countries: Systematic Review Update 2011–2020
Source: Vaccines (Basel). 2024 Nov 23;12(12):1313. doi: 10.3390/vaccines12121313 (PMC11680062; doi:10.3390/vaccines12121313)
Supplement: Supplementary file 1 [file vaccines-12-01313-s001.zip › vaccines-3306848-supplementary.pdf]

Table S1 List of studies excluded on full-text review and reason for exclusion (n=427)

Supplementary material to Shah et al, Integrated approaches for the delivery of maternal and child health services with childhood immunization programs in low- and middle- income countries: systematic review update 2011-2020.

Listed as First Author, Publication Year.

#	Study[a]	Reason for exclusion
1	Aadil 2014	Design-cross-sectional no intervention
2	Abdulraheem 2011	Design-cross-sectional no intervention
3	Abel 2016	Design-cross-sectional no intervention
4	Abuhaloob 2019	Publication-review article
5	Adamu 2019	Intervention-not integration
6	Adamu 2020	Intervention-not integration
7	Adubra 2017	Publication-protocol or abstract
8	Afnan-Holmes 2015	Design-cross-sectional no intervention
9	Agbozo 2017	Publication-conference abstract
10	Agbozo 2018	Design-cross-sectional no intervention
11	Aguilera 2014	Design-cross-sectional no intervention
12	Ahmad 2019	Design-cross-sectional no intervention
13	Aiga 2016	Design-cross-sectional no intervention
14	Aiga 2016	Design-cross-sectional no intervention
15	Aiga 2018	Design-cross-sectional no intervention
16	Ajayi 2013	Design-cross-sectional no intervention
17	Alba 2017	Publication-conference abstract
18	Albaugh 2019	Publication-conference abstract
19	Alhassan 2019	Intervention-not integration
20	Ali 2015	Intervention-not integration
21	Al-Mudhwahi 2015	Design-cross-sectional no intervention
22	Alves 2013	Design-cross-sectional no intervention
23	Amouzou 2012	Design-cross-sectional no intervention
24	Anand 2012	Design-cross-sectional no intervention
25	Andrews 2015	Design-cross-sectional no intervention
26	Antabak 2018	Publication-conference abstract
27	Arima 2013	Design-cross-sectional no intervention
28	Arriola 2016	Population-older than 2YL
29	Asante 2020	Intervention-not integration
30	Asegedew 2019	Intervention-not integration
31	Aslesh 2013	Design-cross-sectional no intervention
32	Assegaai 2017	Publication-conference abstract
33	Assegaai 2018	Intervention-not integration

#	Study[a]	Reason for exclusion
34	Atnafu 2017	Intervention-not integration
35	Ayton 2015	Setting-not LMIC
36	Babaniyi 2013	Intervention-not integration
37	Baker 2017	Publication-review article
38	Balakrishnan 2016	Intervention-not integration
39	Balasubramaniam 2012	Intervention-not integration
40	Baldewsingh 2018	Publication-conference abstract
41	Banerjee 2016	Publication-conference abstract
42	Bano 2015	Population-older than 2YL
43	Baral 2018	Intervention-not integration
44	Barasa 2019	Intervention-not integration
45	Bar-Zeev 2015	Design-cross-sectional no intervention
46	Basaleem 2011	Design-cross-sectional no intervention
47	Bedford 2014	Design-cross-sectional no intervention
48	Bedford 2017	Intervention-not integration
49	Beeson 2011	Publication-opinion
50	Begum 2016	Design-cross-sectional no intervention
51	Belemsaga 2017	Publication-conference abstract
52	Berenson 2016	Setting-not LMIC
53	Berk 2012	Intervention-not integration
54	Bernasconi 2019	Intervention-not integration
55	Besada 2016	Design-cross-sectional no intervention
56	Bhutta 2013	Publication-review article
57	Bhutta 2014	Publication-review article
58	Biemba 2016	Intervention-not integration
59	Binagwaho 2013	Intervention-not integration
60	Binagwaho 2013	Publication-opinion
61	Blaauw 2015	Design-cross-sectional no intervention
62	Bland 2013	Intervention-not integration
63	Blazer 2016	Publication-review article
64	Bluml 2018	Setting-not LMIC
65	Bonfrer 2016	Intervention-not integration
66	Bonner 2011	Intervention-not integration
67	Bosomprah 2016	Intervention-not integration
68	Bradford 2013	Publication-review article
69	Brault 2017	Methods-insufficient info
70	Brew 2019	Publication-review article
71	Bright 2018	Publication-review article
72	Broutet 2013	Population-older than 2YL
73	Brown 2014	Setting-not LMIC

#	Study[a]	Reason for exclusion
74	Brown 2015	Design-cross-sectional no intervention
75	Brown 2016	Intervention-not integration
76	Camponovo 2019	Design-modelling
77	Carlson 2011	Duplicate
78	Carlson 2013	Design-cross-sectional no intervention
79	Cazola 2014	Intervention-not integration
80	CentersforDisease 2013	Design-cross-sectional no intervention
81	Chamla 2014	Publication-review article
82	Chamla 2015	Duplicate
83	Chamla 2015	Publication-opinion
84	Chehab 2016	Design-cross-sectional no intervention
85	Chesoli 2018	Design-cross-sectional no intervention
86	Chi 2013	Publication-opinion
87	Chimpololo 2019	Design-cross-sectional no intervention
88	Chitashvili 2017	Publication-conference abstract
89	Chutiyami 2019	Publication-review article
90	Ciapponi 2017	Publication-review article
91	Cibulskis 2012	Design-cross-sectional no intervention
92	Cleland 2015	Publication-opinion
93	Closser 2014	Design-cross-sectional no intervention
94	Coates 2013	Design-cross-sectional no intervention
95	Coghlan 2014	Intervention-not integration
96	Cohen 2012	Publication-opinion
97	Cohen 2015	Design-cross-sectional no intervention
98	Cohn 2016	Publication-review article
99	Coleman 2011	Setting-not LMIC
100	Craig 2017	Publication-review article
101	Cranmer 2017	Design-cross-sectional no intervention
102	Crawley 2012	Intervention-not integration
103	Creel 2017	Publication-conference abstract
104	Cristia 2015	Intervention-not integration
105	Cristia 2015	Intervention-not integration
106	CruzadodelaVega 2017	Publication-foreign language
107	Cuna 2017	Setting-not LMIC
108	Curry 2013	Design-cross-sectional no intervention
109	Dassah 2015	Population-older than 2YL
110	Deardorff 2018	Publication-review article
111	DeSousa 2012	Design-cross-sectional no intervention
112	DeSousa 2012	Intervention-not integration
113	deSouzaCruz 2017	Publication-review article

#	Study[a]	Reason for exclusion
114	Devaliya 2018	Publication-conference abstract
115	DiAngi 2011	Population-older than 2YL
116	Diese 2016	Design-cross-sectional no intervention
117	Dissieka 2019	Intervention-not integration
118	Doherty 2015	Design-cross-sectional no intervention
119	Dolan 2011	Intervention-not integration
120	Dorton 2016	Population-older than 2YL
121	Druetz 2018	Intervention-not integration
122	Dube 2014	Design-cross-sectional no intervention
123	Duysburgh 2015	Design-cross-sectional no intervention
124	Eng 2013	Design-cross-sectional no intervention
125	Fatiregun 2014	Design-cross-sectional no intervention
126	Fekadu 2016	Intervention-not integration
127	Field 2018	Intervention-not integration
128	Fields 2013	Publication-opinion
129	Findley 2013	Intervention-not integration
130	Findley 2013	Intervention-not integration
131	Fisker 2018	Publication-foreign language
132	Flora 2012	Design-cross-sectional no intervention
133	Fort 2011	Design-cross-sectional no intervention
134	Freeman 2017	Publication-review article
135	Fregnani 2013	Population-older than 2YL
136	Gaffield 2014	Publication-opinion
137	Gagneur 2018	Setting-not LMIC
138	Ganguly 2018	Intervention-not integration
139	Gargano 2012	Design-cross-sectional no intervention
140	Gentile 2015	Design-cross-sectional no intervention
141	George 2017	Publication-opinion
142	Giri 2015	Design-cross-sectional no intervention
143	Glenton 2013	Publication-review article
144	Greenwood 2011	Publication-opinion
145	Griffiths 2011	Publication-opinion
146	Gupta 2013	Design-cross-sectional no intervention
147	Gupta 2016	Intervention-not integration
148	Gupta 2018	Intervention-not integration
149	Haile 2013	Population-older than 2YL
150	Hamadani 2014	Publication-review article
151	Hammanyero 2018	Intervention-not integration
152	Hanson 2018	Design-cross-sectional no intervention
153	Hartman 2012	Publication-conference abstract

#	Study[a]	Reason for exclusion
154	Hasan 2015	Publication-conference abstract
155	Hategeka 2017	Intervention-not integration
156	Hayford 2014	Intervention-not integration
157	Higgins-Steele 2015	Intervention-not integration
158	Himani 2018	Design-cross-sectional no intervention
159	Hindin 2015	Population-older than 2YL
160	Hodges 2011	Population-older than 2YL
161	Honarvar 2012	Population-older than 2YL
162	Horng 2016	Publication-conference abstract
163	Horwood 2012	Design-cross-sectional no intervention
164	Hounton 2015	Design-cross-sectional no intervention
165	Hu 2018	Design-modelling
166	Huang 2011	Design-cross-sectional no intervention
167	Huang 2018	Publication-review article
168	Hutin 2013	Intervention-not integration
169	Ibraheem 2019	Design-cross-sectional no intervention
170	Islam 2017	Publication-conference abstract
171	Iwamoto 2013	Design-cross-sectional no intervention
172	Jaca 2018	Publication-review article
173	Jacob 2015	Design-cross-sectional no intervention
174	Jacobs 2012	Design-cross-sectional no intervention
175	Jalang'o 2017	Design-cross-sectional no intervention
176	Janmohamed 2017	Methods-insufficient info
177	Jawahar 2017	Design-cross-sectional no intervention
178	Jigjidsuren 2019	Design-cross-sectional no intervention
179	JimboSotomayor 2019	Design-cross-sectional no intervention
180	Johri 2013	Design-cross-sectional no intervention
181	Johri 2016	Design-modelling
182	Johri 2019	Intervention-not integration
183	Joubert 2013	Design-cross-sectional no intervention
184	Kamadjeu 2015	Intervention-not integration
185	Kamatsuchi 2019	Publication-review article
186	Kamso 2016	Publication-review article
187	Kamugisha 2018	Intervention-not integration
188	Kanyuuru 2015	Intervention-not integration
189	Karim 2015	Design-cross-sectional no intervention
190	Katibeh 2015	Population-older than 2YL
191	Keats 2017	Publication-review article
192	Keiko 2019	Publication-review article
193	Khan 2013	Intervention-not integration

#	Study[a]	Reason for exclusion
194	Kharbanda 2011	Population-older than 2YL
195	Khuzwayo 2018	Intervention-not integration
196	Kihara 2015	Population-older than 2YL
197	Kimani-Murage 2017	Intervention-not integration
198	Kiplagat 2014	Design-cross-sectional no intervention
199	Koenker 2013	Design-cross-sectional no intervention
200	Kong 2015	Setting-not LMIC
201	Koroma 2019	Publication-conference abstract
202	Kosec 2015	Design-cross-sectional no intervention
203	Koulidiati 2016	Design-cross-sectional no intervention
204	Kubalalika 2019	Intervention-not integration
205	Kupka 2013	Design-modelling
206	Kuppuswamy 2016	Publication-conference abstract
207	LaMontagne 2011	Population-older than 2YL
208	LaMontagne 2014	Population-older than 2YL
209	Latthaphasavang 2019	Intervention-not integration
210	Lemaitre 2019	Setting-not LMIC
211	Levin 2013	Population-older than 2YL
212	Lhamsuren 2012	Design-cross-sectional no intervention
213	Li 2017	Intervention-not integration
214	Linde 2019	Publication-review article
215	Little 2012	Design-cross-sectional no intervention
216	Lori 2019	Design-cross-sectional no intervention
217	Loskutova 2020	Setting-not LMIC
218	Luboga 2016	Intervention-not integration
219	Luman 2012	Publication-opinion
220	Lund 2014	Population-older than 2YL
221	Macharia 2017	Design-modelling
222	MacPhail 2013	Population-older than 2YL
223	Magwood 2018	Publication-review article
224	Magwood 2019	Publication-review article
225	Mahanta 2016	Intervention-not integration
226	Malama 2020	Intervention-not integration
227	Malarcher 2014	Design-cross-sectional no intervention
228	Manjeswori 2012	Design-cross-sectional no intervention
229	Mao 2013	Intervention-not integration
230	Marais 2013	Publication-review article
231	Marin 2011	Population-older than 2YL
232	Masresha 2020	Intervention-not integration
233	Matambo 2019	Design-cross-sectional no intervention

#	Study[a]	Reason for exclusion
234	Matangila 2015	Publication-review article
235	Matangila 2017	Publication-conference abstract
236	Matsubayashi 2011	Design-cross-sectional no intervention
237	Matsuoka 2014	Design-cross-sectional no intervention
238	Mazaba 2019	Intervention-not integration
239	Mazzoni 2016	Setting-not LMIC
240	Mbaeyi 2017	Design-cross-sectional no intervention
241	Mbizvo 2013	Publication-opinion
242	McGlynn 2015	Design-cross-sectional no intervention
243	McNaughton 2019	Design-modelling
244	Mehta 2017	Design-cross-sectional no intervention
245	Mihigo 2015	Publication-opinion
246	Miller 2014	Design-cross-sectional no intervention
247	Min 2020	Publication-conference abstract
248	Mirkuzie 2014	Design-cross-sectional no intervention
249	Mirza 2012	Intervention-not integration
250	Mirza 2012	Intervention-not integration
251	Mody 2012	Design-cross-sectional no intervention
252	Mody 2014	Duplicate
253	Molina-Aguilera 2012	Design-cross-sectional no intervention
254	Monroe 2016	Publication-conference abstract
255	More 2017	Intervention-not integration
256	Morgan 2011	Publication-conference abstract
257	Morris 2018	Intervention-not integration
258	Mortensen 2019	Intervention-not integration
259	Mounier-Jack 2014	Design-cross-sectional no intervention
260	Mounier-Jack 2016	Intervention-not integration
261	Moyer 2016	Design-cross-sectional no intervention
262	Mudany 2015	Intervention-not integration
263	Mudey 2012	Design-cross-sectional no intervention
264	Mugisha 2015	Population-older than 2YL
265	Mulebeke 2019	Intervention-not integration
266	Munk 2019	Publication-review article
267	Munoz 2015	Publication-review article
268	Munoz 2018	Publication-conference abstract
269	Muranjan 2011	Design-cross-sectional no intervention
270	Mutabazi 2017	Publication-review article
271	Mutabazi 2020	Design-cross-sectional no intervention
272	Mutahunga 2017	Publication-conference abstract
273	MutiaNasir 2017	Intervention-not integration

#	Study[a]	Reason for exclusion
274	Mutua 2016	Design-cross-sectional no intervention
275	Mwangome 2012	Design-cross-sectional no intervention
276	Mwingira 2016	Duplicate
277	Mwingira 2016	Methods-insufficient info
278	Najnin 2017	Population-older than 2YL
279	Narasimhan 2017	Publication-opinion
280	Narayanappa 2015	Design-cross-sectional no intervention
281	Ndaba 2019	Intervention-not integration
282	Ndiaye 2011	Intervention-not integration
283	Ndiaye 2012	Intervention-not integration
284	Ndokera 2011	Design-cross-sectional no intervention
285	Ng 2017	Design-modelling
286	Nguyen 2013	Publication-review article
287	Nguyen 2017	Intervention-not integration
288	Nguyen 2019	Publication-conference abstract
289	Nikuze 2017	Publication-conference abstract
290	Nikuze 2017	Publication-conference abstract
291	Njau 2013	Intervention-not integration
292	Nonaka 2012	Design-cross-sectional no intervention
293	Nutman 2013	Publication-review article
294	Nyaku 2017	Design-cross-sectional no intervention
295	Oga 2011	Design-cross-sectional no intervention
296	Ogundele 2018	Intervention-not integration
297	Okeibunor 2016	Intervention-not integration
298	Okeibunor 2018	Design-cross-sectional no intervention
299	Okeibunor 2018	Publication-review article
300	Okenwa 2020	Design-cross-sectional no intervention
301	Oktaria 2017	Intervention-not integration
302	Oku 2017	Design-cross-sectional no intervention
303	Okwo-Bele 2012	Publication-opinion
304	Oladele 2012	Design-cross-sectional no intervention
305	O'Meara 2011	Design-cross-sectional no intervention
306	Onimawo 2013	Design-cross-sectional no intervention
307	Oryema 2017	Design-cross-sectional no intervention
308	Osaki 2013	Design-cross-sectional no intervention
309	Osaki 2015	Design-cross-sectional no intervention
310	Otieno-Masaba 2013	Intervention-not integration
311	Otsuka-Ono 2019	Setting-not LMIC
312	Oyo-Ita 2016	Publication-review article
313	Ozawa 2018	Design-cross-sectional no intervention

#	Study[a]	Reason for exclusion
314	Padmadas 2017	Publication-opinion
315	Palmer 2013	Design-cross-sectional no intervention
316	Pardhi 2020	Design-cross-sectional no intervention
317	Partapuri 2012	Publication-review article
318	Patchala 2016	Design-cross-sectional no intervention
319	Patel 2012	Intervention-not integration
320	Pathirana 2015	Population-older than 2YL
321	Paul 2014	Population-older than 2YL
322	Penny 2011	Population-older than 2YL
323	Perry 2017	Publication-review article
324	Pezzoli 2017	Design-cross-sectional no intervention
325	Philips 2019	Setting-not LMIC
326	Philipson 2018	Publication-conference abstract
327	Picolo 2019	Methods-insufficient info
328	Poulos 2016	Publication-conference abstract
329	Prinja 2017	Intervention-not integration
330	Quentin 2012	Population-older than 2YL
331	R 2019	Design-cross-sectional no intervention
332	Raesima 2015	Population-older than 2YL
333	Raghunathan 2017	Intervention-not integration
334	Rah 2014	Design-cross-sectional no intervention
335	Rahman 2016	Intervention-not integration
336	Rawat 2015	Intervention-not integration
337	Reid 2020	Publication-review article
338	RemundinideLima 2016	Design-cross-sectional no intervention
339	Restrepo-Mendez 2016	Publication-review article
340	Ricca 2014	Intervention-not integration
341	Ropero-Alvarez 2012	Publication-opinion
342	RoperoAlvarez 2017	Publication-review article
343	Rossier 2014	Publication-opinion
344	Ruducha 2017	Publication-review article
345	Ryman 2012	Design-cross-sectional no intervention
346	Saggurti 2018	Intervention-not integration
347	Salam 2014	Intervention-not integration
348	Salam 2019	Publication-review article
349	Saleem 2017	Design-cross-sectional no intervention
350	Sarveswaran 2019	Design-cross-sectional no intervention
351	Schouten 2012	Publication-conference abstract
352	Schuchat 2012	Publication-opinion

#	Study[a]	Reason for exclusion
353	Senegal National Agency of Statistics and Demography 2013	Design-cross-sectional no intervention
354	Sesay 2015	Design-cross-sectional no intervention
355	Sexton 2011	Publication-review article
356	Shepard 2012	Intervention-not integration
357	Shewade 2013	Design-cross-sectional no intervention
358	Sibomana 2018	Intervention-not integration
359	Silumbwe 2017	Publication-review article
360	Silvestre 2018	Intervention-not integration
361	Sinunu 2014	Intervention-not integration
362	Smith 2018	Publication-review article
363	Sobel 2011	Design-cross-sectional no intervention
364	Soeung 2012	Design-cross-sectional no intervention
365	Solon 2018	Publication-conference abstract
366	Sondaal 2016	Publication-review article
367	Speizer 2013	Design-cross-sectional no intervention
368	Sridhar 2014	Publication-review article
369	Stanback 2017	Publication-opinion
370	Suthar 2012	Publication-opinion
371	Suwantika 2020	Design-modelling
372	Tafere 2018	Population-older than 2YL
373	Taqi 2020	Design-cross-sectional no intervention
374	Taylor 2019	Publication-review article
375	Teferi 2014	Design-cross-sectional no intervention
376	Theiss-Nyland 2016	Publication-review article
377	Theiss-Nyland 2016	Design-cross-sectional no intervention
378	Theiss-Nyland 2016	Design-cross-sectional no intervention
379	Theiss-Nyland 2019	Intervention-not integration
380	Thompson 2019	Publication-conference abstract
381	Thwing 2017	Intervention-not integration
382	Titmus 2018	Setting-not LMIC
383	Tohme 2017	Intervention-not integration
384	Torres-Rueda 2016	Population-older than 2YL
385	Tsu 2014	Population-older than 2YL
386	Tumwesigye 2016	Design-cross-sectional no intervention
387	Ubajaka 2012	Design-cross-sectional no intervention
388	Uddin 2012	Intervention-not integration
389	Uddin 2016	Design-cross-sectional no intervention
390	Uneke 2016	Publication-review article
391	Upadhyay 2017	Design-cross-sectional no intervention

#	Study[a]	Reason for exclusion
392	Velleman 2013	Design-cross-sectional no intervention
393	Verguet 2013	Design-cross-sectional no intervention
394	Verguet 2016	Publication-review article
395	Vijayaraghavan 2012	Design-cross-sectional no intervention
396	Villadsen 2016	Intervention-not integration
397	Vince 2014	Design-cross-sectional no intervention
398	Wagner 2018	Intervention-not integration
399	Wallace 2012	Design-cross-sectional no intervention
400	Wallace 2012	Publication-review article
401	Wallace 2017	Intervention-not integration
402	Wallace 2018	Design-cross-sectional no intervention
403	Wallace 2019	Intervention-not integration
404	Watson-Jones 2016	Population-older than 2YL
405	Webb 2018	Publication-review article
406	Wei 2012	Design-cross-sectional no intervention
407	Wei 2012	Design-cross-sectional no intervention
408	Wesevich 2016	Intervention-not integration
409	Wiegert 2014	Design-cross-sectional no intervention
410	Wiesen 2016	Design-cross-sectional no intervention
411	Wiesen 2016	Design-cross-sectional no intervention
412	Wilson 2019	Publication-conference abstract
413	Woldesenbet 2015	Design-cross-sectional no intervention
414	Woldesenbet 2015	Duplicate
415	Woodring 2017	Publication-review article
416	Woolley 2018	Intervention-not integration
417	Wu 2017	Design-cross-sectional no intervention
418	Wu 2018	Intervention-not integration
419	Wu 2018	Intervention-not integration
420	Xeuatvongsa 2016	Intervention-not integration
421	Yonghao 2017	Design-cross-sectional no intervention
422	YugbareBelemsaga 2017	Publication-conference abstract
423	ZegersDeBeyl 2014	Intervention-not integration
424	Zeng 2019	Design-modelling
425	Zeng 2019	Intervention-not integration
426	Zewdie 2016	Design-cross-sectional no intervention
427	Zhang 2019	Design-cross-sectional no intervention
